# Periapical abscess precipitating diabetic ketoacidosis and reversible sepsis-induced cardiomyopathy: a case report

**DOI:** 10.3389/fendo.2026.1853265

**Published:** 2026-07-15

**Authors:** Jixia Jin, Lian Liao, Yinhang Ren, Guiyun Li, Yuyang Qiu

**Affiliations:** Department of Emergency, The Second People’s Hospital of Guiyang (Jinyang Hospital)/The Affiliated Jinyang Hospital of Guizhou Medical University, Guiyang, China

**Keywords:** case report, diabetic ketoacidosis, odontogenic infection, sepsis, sepsis-induced cardiomyopathy

## Abstract

**Background:**

Diabetic ketoacidosis (DKA) is a life-threatening acute complication of diabetes mellitus, most commonly precipitated by infection. Patients with diabetes are at a significantly elevated risk for severe infections and sepsis. Odontogenic infections represent a prevalent but potentially overlooked source of sepsis in this population, given the established bidirectional relationship between diabetes and periodontitis.

**Case presentation:**

A 57-year-old female with poorly controlled type 2 diabetes presented with a one-week history of left jaw pain and one day of severe dyspnea and lethargy. Examination revealed left submandibular swelling, Kussmaul respirations, and lethargy. Laboratory findings confirmed severe DKA (pH 7.04, β-hydroxybutyrate 7.19 mmol/L) and sepsis (leukopenia, procalcitonin 2.34 ng/mL). Echocardiography demonstrated severe left ventricular systolic dysfunction (ejection fraction 36%) with normal cardiac biomarkers, consistent with sepsis-induced cardiomyopathy (SICM). Imaging identified a left submandibular abscess. Management involved prompt sepsis bundle implementation, including early empiric antibiotics (amoxicillin-clavulanate and tinidazole), aggressive fluid resuscitation, and concurrent intravenous insulin infusion for DKA, alongside proactive electrolyte repletion. Multidisciplinary source control was achieved. Outcomes: The patient showed rapid clinical improvement. Metabolic acidosis and ketosis resolved. Infection markers normalized. Remarkably, follow-up echocardiography on day 14 demonstrated complete recovery of cardiac function (ejection fraction 72%). She was discharged after 15 days and remained well at one-month follow-up.

**Conclusions:**

This case underscores that odontogenic infection can be a potent trigger for life-threatening DKA and septic shock with multi-organ dysfunction in diabetic patients. It highlights the critical importance of thorough physical examination, including oral cavity inspection, in the evaluation of sepsis or DKA. The presentation and complete reversibility of SICM reinforce that treatment must focus on correcting the underlying septic and metabolic insults. Early multidisciplinary management is essential for optimal outcomes.

## 1 Introduction

Diabetic ketoacidosis (DKA) remains a serious acute complication of diabetes mellitus, characterized by hyperglycemia, ketonemia, and metabolic acidosis (1, 2). Infection is the most common precipitating factor, accounting for 30-50% of cases (3, 4). Patients with diabetes, particularly those with poor glycemic control, are at a markedly increased risk for severe infections and sepsis, with an estimated sepsis risk up to six times higher than that of the non-diabetic population (5, 6).

The interplay between diabetes and sepsis is complex and bidirectional. Hyperglycemia impairs innate immune functions, including neutrophil chemotaxis and phagocytosis, while the pro-inflammatory cytokine surge in sepsis exacerbates insulin resistance and disrupts glucose homeostasis, creating a vicious cycle that can rapidly lead to multi-organ dysfunction (5, 7–9). Sepsis-induced cardiomyopathy (SICM), a reversible form of myocardial dysfunction often characterized by biventricular dilation and reduced ejection fraction in the absence of primary cardiac ischemia, is a recognized complication that further complicates management (10, 11).

Odontogenic infections, such as periapical or periodontal abscesses, are prevalent yet potentially under-recognized sources of sepsis. The intimate association between periodontitis and diabetes is well-established, with each disease exacerbating the other (12). However, the progression from a localized dental infection to fulminant DKA with multi-organ failure represents a critical clinical scenario that demands heightened awareness. We present a detailed case that exemplifies this dangerous progression and discusses the pertinent pathophysiology and management principles. The patient’s written informed consent has been obtained for the publication of this case report and the accompanying images. Ethical approval by the Institutional Review Board of the Affiliated Jinyang Hospital of Guizhou Medical University (Approval No. JYYY-2026-WZ-01).

## 2 Case description

A 57-year-old female presented to the emergency department with a one-week history of left jaw pain and swelling, followed by 24 hours of progressive dyspnea, profound weakness, and lethargy. Her past medical history was significant for hypertension and poorly controlled type 2 diabetes mellitus (T2DM), the latter evidenced by an admission hemoglobin A1c of 10.2%. She also reported a history of suspected coronary artery disease.

On physical examination, the patient was lethargic and exhibited Kussmaul respirations (rate 44/min). Vital signs included tachycardia (125 bpm) and a blood pressure of 150/84 mmHg. A tender, fluctuant 6 × 5 cm² mass was palpable in the left submandibular region.

Initial laboratory evaluation confirmed a severe metabolic crisis: arterial blood gas revealed pH 7.04, bicarbonate 1.9 mmol/L, and an elevated anion gap. Serum β-hydroxybutyrate was markedly elevated at 7.19 mmol/L, and blood glucose was 14.4 mmol/L, establishing the diagnosis of severe DKA. Sepsis was concurrently diagnosed based on leukopenia (3.4×10^9^/L), elevated procalcitonin (2.34 ng/mL), hs-CRP 26.66 mg/L, and a Sequential Organ Failure Assessment (SOFA) score of 4. Serum troponin T remained undetectable (<0.02 ng/mL), and serial electrocardiograms showed no acute ischemic changes, effectively ruling out acute coronary syndrome. Bedside transthoracic echocardiography demonstrated globally reduced left ventricular systolic function with an ejection fraction (LVEF) of 36%, consistent with SICM. Hemodynamic assessment on admission showed a mean arterial pressure of 78 mmHg, and the patient remained stable without requiring vasopressor or inotropic support; initial serum lactate was 2.8 mmol/L. Serial arterial blood gas analyses revealed progressive resolution of metabolic acidosis, with pH improving from 7.04 at presentation to 7.31 at 6 hours and 7.38 at 24 hours, alongside a concurrent decline in β-hydroxybutyrate from 7.19 mmol/L to 2.1 mmol/L. No vasoactive agents were needed throughout hospitalization. A neck computed tomography scan identified a left submandibular abscess as the likely septic focus (**Figure 1**). The Acute Physiology and Chronic Health Evaluation II (APACHE II) score within 24 hours of admission was 21, indicating critical illness with a high risk of death. Overall oral hygiene was poor, with generalized calculus deposition and multiple carious teeth, consistent with long-standing neglect of dental care. The periapical abscess was attributed to deep dental caries involving tooth 48, with poor oral hygiene and uncontrolled diabetes serving as major predisposing local and systemic factors.

**Figure 1:**
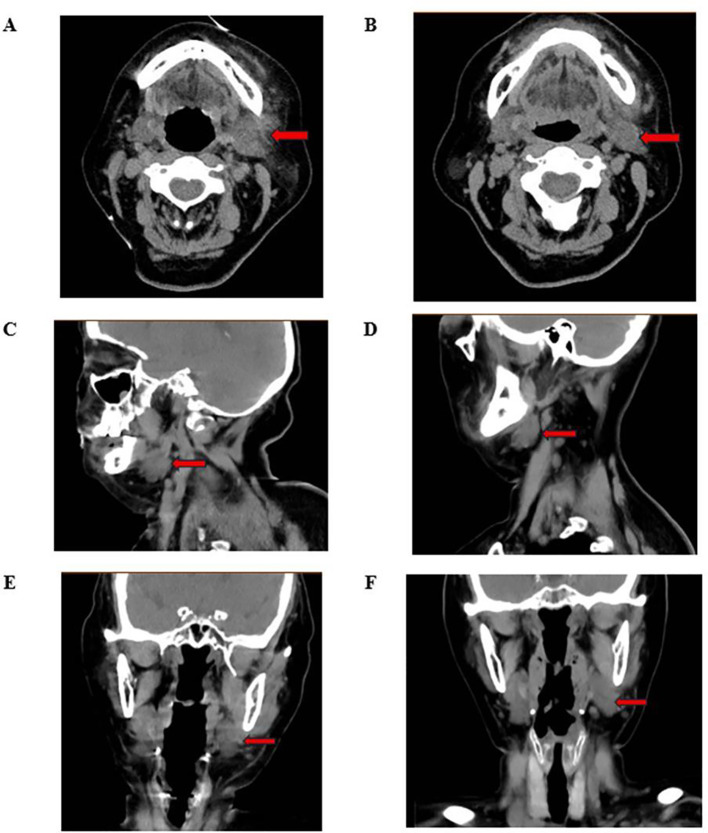
Comparative neck CT imaging before and after treatment. **(A)** Axial view on admission shows an enlarged left submandibular gland (arrow), suggestive of an inflammatory lesion. **(B)** Axial view at the same level after 2 weeks of treatment demonstrates a significant reduction in the size of the left submandibular gland (arrow). **(C)** Sagittal view on admission reveals enlargement of the left submandibular gland (arrow), consistent with inflammatory change. **(D)** Follow-up sagittal view after 2 weeks shows marked decrease in glandular size (arrow). **(E)** Coronal view on admission highlights the enlarged left submandibular gland (arrow). **(F)** Coronal view after 2 weeks of therapy confirms interval reduction in gland size (arrow).

### 2.1 Management and clinical course

Immediate management adhered to sepsis and DKA resuscitation bundles. Within the first hour, empiric broad-spectrum antibiotic therapy with intravenous amoxicillin-clavulanate and tinidazole was initiated. Aggressive fluid resuscitation with crystalloids was undertaken. For DKA, a continuous intravenous insulin infusion at 0.1 U/kg/h was commenced concurrently with fluid therapy to rapidly address the insulin deficiency and ketosis. The patient developed significant hypokalemia (K^+^ nadir 2.85 mmol/L) during treatment, which was proactively managed with potassium supplementation. A multidisciplinary consultation with oral and maxillofacial surgery was obtained for definitive source control.

Following consultation with the Department of Oral and Maxillofacial Surgery, physical examination revealed facial asymmetry with mild soft tissue swelling and tenderness over the left buccal region. Mouth opening was adequate with a normal jaw opening pattern. Intraoral examination identified the residual root of tooth 48. Teeth 41 and 42 exhibited Grade II mobility. Purulent discharge was evident surrounding the root of tooth 48. No fluctuance was palpated in the submandibular or buccal regions. Local skin temperature was not elevated, and erythema was absent. The working diagnosis was a periapical abscess, with submandibular space infection considered in the differential. Continuation of aggressive antibiotic therapy was recommended. Culture of oral secretion subsequently identified *Pseudomonas aeruginosa*, a non−multidrug−resistant strain. Antimicrobial susceptibility testing confirmed sensitivity to the empiric regimen (amoxicillin–clavulanate and tinidazole); therefore, no antibiotic modification was required. The patient responded positively to the initial antimicrobial regimen, with a steady decline in procalcitonin levels, obviating the need for escalation to broader−spectrum agents such as carbapenems. Following resolution of the hyperglycemic crisis, her glycemic control was transitioned from intravenous insulin to a structured multiple daily injection insulin regimen (basal−bolus). Early aggressive fluid resuscitation, a cornerstone of both sepsis and diabetic ketoacidosis management, was administered. After timely correction of metabolic acidosis and adequate control of the inflammatory response, the patient did not meet criteria for inotropic support.

### 2.2 Outcome and follow-up

The clinical course showed consistent improvement. A follow-up echocardiogram performed on hospital day 14 revealed complete normalization of cardiac function, with the LVEF recovering to 72%. All parameters of metabolic acidosis, renal function, and infection markers resolved (**Figure 1**). The patient was discharged after a 15-day hospitalization (**Table 1**). At a one-month post-discharge follow-up, she reported a complete return to her baseline functional status without residual symptoms. Her glycemic control remained stable on the adjusted insulin therapy, confirming a favorable and sustained recovery. At the one-month follow-up, the mobility of teeth 41 and 42 had significantly improved from Grade II to Grade I, and no further purulent discharge was noted.

**Table 1:** Timeline of clinical presentation, interventions, and outcomes.

Time point	Event/intervention	Key clinical data
1 week prior to admission	Onset of left jaw pain and swelling	Patient initially ignored symptoms; no medical consultation
24 hours prior to admission	Progressive dyspnea, profound weakness, lethargy	Family noticed worsening; decision to seek emergency care
Day 0 (Admission)	Emergency department presentation	Vitals: HR 125 bpm, BP 150/84 mmHg, RR 44/min (Kussmaul) Physical exam: Lethargic, left submandibular fluctuant mass (6 × 5 cm²) ABG: pH 7.04, HCO_3_^-^ 1.9 mmol/L Laboratory: Glucose 14.4 mmol/L, β-hydroxybutyrate 7.19 mmol/L, WBC 3.4×10^9^/L, PCT 2.34 ng/mL, hs-CRP 26.66 mg/L, troponin T <0.02 ng/mL Echocardiography: LVEF 36% (global hypokinesis) CT neck: Left submandibular abscess Scores: SOFA 4, APACHE II 21 (predicted mortality 38.91%)
Day 0 (First hour)	Initiation of sepsis and DKA bundles	• IV amoxicillin-clavulanate + tinidazole • IV fluid resuscitation (crystalloids) • IV insulin infusion 0.1 U/kg/h • Potassium supplementation (nadir K^+^ 2.85 mmol/L)
Day 1	Multidisciplinary consultation (Oral & Maxillofacial Surgery, Clinical Pharmacy Department)	Intraoral exam: residual root of tooth 48, purulent discharge; diagnosis: periapical abscess; no surgical drainage required due to good response to antibiotics
Day 3	Culture result	Oral secretion culture: *Pseudomonas aeruginosa* (non-MDR)
Day 7	Clinical improvement	• Resolution of tachypnea and lethargy • Downward trend of PCT and inflammatory markers • Transition from IV insulin to basal-bolus subcutaneous insulin
Day 14	Follow-up echocardiography	LVEF 72% (complete normalization)
Day 15	Discharge	• Normal metabolic profile • Stable on insulin regimen • Discharged home
1 month post-discharge	Outpatient follow-up	• Asymptomatic, returned to baseline functional status • Glycemic control stable on adjusted insulin therapy

ABG, arterial blood gas; APACHE II, Acute Physiology and Chronic Health Evaluation II; BP, blood pressure; CT, computed tomography; DKA, diabetic ketoacidosis; HR, heart rate; hs-CRP, high-sensitivity C-reactive protein; IV, intravenous; LVEF, left ventricular ejection fraction; MDR, multidrug-resistant; PCT, procalcitonin; RR, respiratory rate; SOFA, Sequential Organ Failure Assessment; WBC, white blood cell count. Conversion factors: glucose (mg/dL to mmol/L: divide by 18); β-hydroxybutyrate (mg/dL to mmol/L: divide by 10.4). Predicted mortality based on APACHE II score calculated using standard formula.

## 3 Discussion

This case provides a compelling and clinically significant illustration of how a localized odontogenic infection can serve as the inciting event for a cascade of severe systemic complications in a vulnerable host with poorly controlled T2DM. It underscores the critical “malignant synergy” between sepsis and DKA—a bidirectional pathological relationship where each condition exacerbates the other, leading to rapid clinical deterioration (7, 13). The initial dental abscess provoked a profound systemic inflammatory response, triggering a massive release of counter-regulatory hormones (e.g., cortisol, catechilamines, glucagon). This hormonal surge induced severe insulin resistance and accelerated lipolysis, culminating in the life-threatening DKA observed at presentation (1–4, 14). Concurrently, the hyperglycemic, acidotic, and hyperosmolar environment of DKA is known to impair multiple arms of the innate immune system, including neutrophil chemotaxis, phagocytosis, and complement function, thereby crippling the host’s ability to contain the initial infection and facilitating the progression to septic shock (7, 15). This vicious cycle of metabolic dysfunction and immune paralysis is a hallmark of diabetic sepsis and was vividly demonstrated in this patient’s presentation with multi-organ failure.

A paramount learning point from this case is the indispensable need for a meticulous search for an infectious source in any patient presenting with DKA. Infections precipitate 30-50% of DKA episodes, and their identification is crucial for targeted therapy (1, 2, 14). Identifying the infectious trigger in patients presenting with diabetic ketoacidosis remains clinically challenging, as conventional sepsis screening tools have limited predictive accuracy in this setting (16). This case emphasizes that this search must routinely include a thorough examination of the oral cavity and dentition. The association between periodontitis and diabetes is well-established and bidirectional; diabetes increases the risk and severity of periodontitis, and chronic periodontal inflammation can adversely affect glycemic control (17, 18). Diabetes mellitus has been identified as an independent predictor of severe odontogenic infections, with diabetic patients exhibiting significantly higher infection severity scores compared to non-diabetic individuals (19). Our patient’s markedly elevated HbA1c (10.2%) reflects long-term hyperglycemia, which undoubtedly contributed to a pro-inflammatory oral microenvironment, increased vascular permeability, and impaired local defense mechanisms, setting the stage for an aggressive odontogenic infection (12, 18). Severe septic progression from odontogenic infection is relatively rare, occurring in approximately 3% of hospitalized patients with odontogenic infections (20). Clinicians must be vigilant that seemingly minor dental complaints in diabetic patients can mask a potential source of severe sepsis.

The presence of acute, severe myocardial dysfunction in this setting is characteristic of Sepsis-Induced Cardiomyopathy (SICM). SICM is diagnosed by the onset of reversible biventricular systolic and/or diastolic dysfunction in the setting of severe infection, typically in the absence of other clear causes like acute coronary syndrome (10, 21). Its pathophysiology is multifactorial, involving direct myocardial depression by inflammatory cytokines (e.g., TNF-α, IL-1β), mitochondrial dysfunction, autonomic dysregulation, and microcirculatory disturbances (10, 11, 21). The complete normalization of the LVEF from 36% to 72% following source control and correction of metabolic derangements in our patient is a classic and powerful demonstration of SICM’s reversibility. This outcome reinforces the principle that the cornerstone of SICM management is the aggressive treatment of the underlying sepsis and associated metabolic abnormalities, rather than the reflexive use of inotropic agents, which may increase myocardial oxygen demand without addressing the primary insult (10, 11, 21).

In this patient, the possibility of acute coronary syndrome was excluded by undetectable high-sensitivity troponin T and serial electrocardiograms showing no ischemic changes. Myocarditis was considered less likely given the absence of fever prior to presentation, lack of typical electrocardiographic findings (e.g., diffuse ST-segment elevations), and the rapid normalization of cardiac function following sepsis control. Stress-induced (Takotsubo) cardiomyopathy was also considered; however, the pattern of global hypokinesis rather than apical ballooning, along with the temporal association with septic shock, favored sepsis-induced cardiomyopathy over Takotsubo. Thus, the diagnosis of SICM was established as the most consistent explanation.

The management of this complex case integrated several key, evidence-based principles. First, the timely implementation of the sepsis resuscitation bundle, including early appropriate antibiotics and fluid resuscitation, was fundamental (22). The combination of amoxicillin–clavulanate with metronidazole has been shown to be effective as empiric therapy for odontogenic infections in both diabetic and non-diabetic patients (23). The choice of amoxicillin-clavulanate and tinidazole provided effective empiric coverage against the typical polymicrobial oropharyngeal flora, including aerobic streptococci and anaerobic bacteria, and the favorable clinical response validated this choice (24). Second, the concurrent initiation of fluid resuscitation and a low-dose continuous insulin infusion for DKA followed current consensus guidelines, which prioritize volume expansion while promptly addressing insulin deficiency to halt ketogenesis (1–3, 14). The anticipated and proactively managed hypokalemia highlights the importance of vigilant electrolyte monitoring. Third, the transition to a basal-bolus insulin regimen upon DKA resolution is crucial for achieving stable glycemic control in the post-critical phase and preventing rebound hyperglycemia (14). Finally, early multidisciplinary involvement for source control, was instrumental in breaking the cycle of infection and inflammation.

As a single-case report, the findings presented here are not generalizable. Cardiac evaluation was limited to transthoracic echocardiography; advanced imaging modalities such as cardiac magnetic resonance were not performed, which could have provided additional insight into myocardial tissue characterization and further excluded alternative diagnoses. Clinical photographs of the oral and extraoral lesions were not obtained due to institutional policy and patient privacy considerations. Cone beam computed tomography (CBCT) was not performed; the diagnosis of periapical abscess was based on clinical examination and conventional non−contrast CT of the neck, which sufficiently identified the inflammatory focus. Nevertheless, the complete functional recovery of left ventricular ejection fraction following resolution of the septic and metabolic insults strongly supports the diagnosis of reversible sepsis-induced cardiomyopathy.

## 4 Patient perspective

Upon reflection, the patient described her experience as “terrifying and enlightening.” She reported that she had initially dismissed the jaw pain as a minor dental issue. “I never imagined a tooth problem could make me so sick that I couldn’t breathe, “ she stated. The rapid progression to severe illness was frightening for her and her family. She expressed profound gratitude for the coordinated efforts of the intensive care, endocrinology, and dental surgery teams, whose clear communication helped her understand the serious link between her diabetes, the infection, and her heart function. Following recovery, she acknowledged a significant shift in her attitude towards health management: “This was a major wake-up call. I now understand that taking care of my diabetes isn’t just about checking my blood sugar; it also means taking care of my teeth and listening to my body. I am committed to keeping both under better control.” Her perspective underscores the profound personal impact of such an illness and highlights the importance of patient education on the systemic connections between oral health and chronic disease management.

## 5 Conclusions

In conclusion, this report highlights odontogenic infection as a potent and sometimes overlooked trigger for life-threatening DKA and septic shock in patients with diabetes. It reinforces the critical importance of a comprehensive physical examination to identify occult septic foci and vividly illustrates the dramatic reversibility of sepsis-induced cardiomyopathy with timely, aggressive, and multidisciplinary care aimed at the underlying causes. For patients with diabetes, this case underscores that optimal long-term management must extend beyond glycemic metrics to include proactive dental care, as maintaining oral health is a vital component of preventing severe systemic complications.

## Data Availability

Data sharing does not apply to this article, as no datasets were generated or analyzed during the current study.
